# Combined Treatment with Cryptocaryone and Ultraviolet C Promotes Antiproliferation and Apoptosis of Oral Cancer Cells

**DOI:** 10.3390/ijms23062981

**Published:** 2022-03-10

**Authors:** Sheng-Chieh Wang, Hsun-Shuo Chang, Jen-Yang Tang, Ammad Ahmad Farooqi, Yun-Tzu Kuo, Yan-Der Hsuuw, Jai-Wei Lee, Hsueh-Wei Chang

**Affiliations:** 1 Department of Biomedical Science and Environmental Biology, Ph.D. Program in Life Sciences, College of Life Sciences, Kaohsiung Medical University, Kaohsiung 80708, Taiwan; u107851101@gap.kmu.edu.tw (S.-C.W.); u106023026@kmu.edu.tw (Y.-T.K.); 2 Graduate Institute of Natural Products, College of Pharmacy, Kaohsiung Medical University, Kaohsiung 80708, Taiwan; hschang@kmu.edu.tw; 3 School of Pharmacy, College of Pharmacy, Kaohsiung Medical University, Kaohsiung 80708, Taiwan; 4 School of Post-Baccalaureate Medicine, Kaohsiung Medical University, Kaohsiung 80708, Taiwan; reyata@kmu.edu.tw; 5 Department of Radiation Oncology, Kaohsiung Medical University Hospital, Kaohsiung 80708, Taiwan; 6 Institute of Biomedical and Genetic Engineering (IBGE), Islamabad 54000, Pakistan; farooqiammadahmad@gmail.com; 7 Department of Tropical Agriculture and International Cooperation, National Pingtung University of Science and Technology, Pingtung 912301, Taiwan; hsuuw@mail.npust.edu.tw; 8 Center for Cancer Research, Kaohsiung Medical University, Kaohsiung 80708, Taiwan; 9 Institute of Medical Science and Technology, National Sun Yat-Sen University, Kaohsiung 80424, Taiwan; 10 Department of Medical Research, Kaohsiung Medical University Hospital, Kaohsiung 80708, Taiwan

**Keywords:** cryptocaryone, ultraviolet C, oral cancer, combined effect, apoptosis, DNA damage

## Abstract

Cryptocaryone (CPC) was previously reported as preferential for killing natural products in oral cancer cells. However, its radiosensitizing potential combined with ultraviolet C (UVC) cell killing of oral cancer cells remains unclear. This study evaluates the combined anti-proliferation effect and clarifies the mechanism of combined UVC/CPC effects on oral cancer cells. UVC/CPC shows higher anti-proliferation than individual and control treatments in a low cytotoxic environment on normal oral cells. Mechanistically, combined UVC/CPC generates high levels of reactive oxygen species and induces mitochondrial dysfunction by generating mitochondrial superoxide, increasing mitochondrial mass and causing the potential destruction of the mitochondrial membrane compared to individual treatments. Moreover, combined UVC/CPC causes higher G2/M arrest and triggers apoptosis, with greater evidence of cell cycle disturbance, annexin V, pancaspase, caspases 3/7 expression or activity in oral cancer cells than individual treatments. Western blotting further indicates that UVC/CPC induces overexpression for cleaved types of poly (ADP-ribose) polymerase and caspase 3 more than individual treatments. Additionally, UVC/CPC highly induces γH2AX and 8-hydroxy-2’-deoxyguanosine adducts as DNA damage in oral cancer cells. Taken together, CPC shows a radiosensitizing anti-proliferation effect on UVC irradiated oral cancer cells with combined effects through oxidative stress, apoptosis and DNA damage.

## 1. Introduction

Oral cancer is a high morbidity-causing cancer worldwide [1,2], which causes high mortality at the same time [3]. Oral cancer is highly prevalent in several countries with the increased popularity of betel nut chewing, alcohol consumption, and tobacco smoking [4]. The incidence and death rate of oral cancer increases every year for both genders [5]. Therefore, it is vital to cure oral cancer by developing more effective treatments.

Combined treatment of anticancer agents with radiotherapy is a common strategy in oral cancer therapy [6,7]. However, this combined treatment commonly shows side effects on normal tissues because of the cytotoxicity of common radiosensitizers. Accordingly, radiosensitizing agents with a preferential killing ability are expected to reduce side effects on normal cells.

X-rays are commonly used for ionizing radiotherapy. Alternatively, ultraviolet C (UVC; ranging from 200 to 280 nm), providing non-ionizing radiation, may have a potential in terms of radiotherapy in the treatment of cancer. Accumulated evidence shows that combined treatment of anticancer agents with UVC also improves the anticancer effect. For example, a combined treatment of cisplatin/UVC enhances anti-proliferation against colorectal cancer cells [8]. In addition, combined treatments such as ethyl acetate *Nepenthes* extract/UVC [9] and sulfonyl chromen-4-ones/UVC [10] synergistically inhibit the proliferation of oral cancer cells. UVC has been used in clinical trials but mainly focuses on room decontamination [11]. Although UVC exhibits low penetration ability [12], the anticancer effects of UVC have been applied in several animal studies [13,14,15]. The application of UVC to clinical applications is under development.

*Cryptocarya concinna* methanol extract (MECCrt) shows antioral cancer effects and triggers apoptosis in association with the overexpression of reactive oxygen species (ROS) and mitochondrial membrane potential (MMP) depletion [16]. The natural product crude extract MECCrt was used for combined treatment with UVC irradiation in oral cancer cells, and it enhances preferential killing and apoptosis of oral cancer cells [17]. Therefore, it warrants a comprehensive investigation of whether the responsible bioactive compound in the crude extract MECCrt causes preferential killing and/or anti-proliferation in a combined treatment of MECCrt/UVC.

Cryptocaryone (CPC) was identified as one of the main bioactive compounds in MECCrt [18,19]. Although the anticancer effect of this dihydrochalcone CPC was reported in some cancer types such as murine leukemia [20] and prostate cancer [19], few studies have addressed its preferential killing effect on cancer cells. Recently [21], we found that CPC exhibited a preferential killing ability regarding oral cancer cells (Ca9-22 and CAL 27) through ROS induction. Still, CPC shows low cytotoxicity to normal oral (HGF-1) cells. However, the effect of a combined treatment of UVC with CPC (UVC/CPC) in oral cancer therapy remains unclear.

The present study focuses on improving anti-proliferation in oral cancer cells by a combined UVC/CPC treatment. It explores its potential mechanisms regarding oxidative stress-associated changes such as apoptosis and DNA damage.

## 2. Results

### 2.1. Cooperative Antiproliferation of Oral Cancer Cells by UVC/CPC Treatment

**Figure 1:**
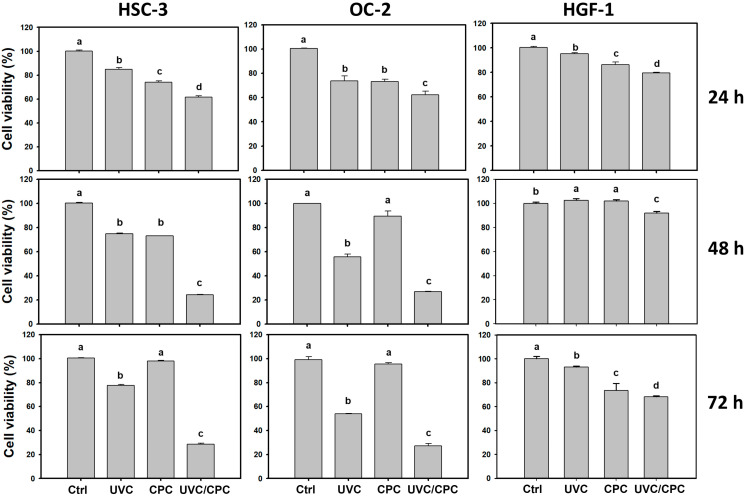
Cell viability change of oral cancer and normal oral cells following UVC or/and CPC treatments. Oral cancer (HSC-3 and OC-2) and normal oral (HGF-1) cells were arranged to treat with vehicle control, UVC (10 J/m^2^), CPC (5 μg/mL), and UVC/CPC (10 J/m^2^ and 5 μg/mL). A 24, 48, and 72 h MTS assay measured cell viability. Based on multiple comparisons after Tukey’s HSD Tests, different tests labeled with different top letters differed significantly (*p* < 0.05). Data is presented as means ± SD (*n* = 3). For example, OC-2 cells at 24 h MTS, UVC and CPC showing “b, b” with the overlapping letter ”b” indicated a non-significant difference between each other. Control, UVC, and UVC/CPC in OC-2 cells showing “a, b, c” without overlapping letters indicated a significant difference between each other.

For UVC and CPC alone, the IC_50_ values for HSC-3 cells were 35 J/m^2^ and 15 μg/mL, while for OC-2 cells they were 17 J/m^2^ and 13 μg/mL, respectively. After testing different conditions, the optimal condition for UVC and CPC (10 J/m^2^ and 5 μg/mL), around 70–80% viability at 24 h MTS assay, were chosen for single and combined treatments of two oral cancer (HSC-3 and OC-2) and one normal oral (HGF-1) cell line (Figure 1). The viability of combined treatment (UVC/CPC)-treated HSC-3 cells (62%) was lower than that of single treatment (UVC: 85% or CPC: 74%). Likewise, the viability of UVC/CPC-treated OC-2 cells (62.1%) was lower than that of a single treatment (UVC: 73.8% or CPC: 73.1%). In contrast, the cell viabilities for UVC, CPC, and UVC/CPC in normal oral cells were 95.3, 86.3, and 79.4, respectively. Moreover, UVC/CPC in normal oral cells showed a lower cytotoxicity effect than oral cancer cells.

Moreover, the same condition was applied to 48 and 72 h MTS assays. For 48 h viability, the cell viability for the UVC/CPC of HSC-3 and OC-2 cells was 24.2% and 26.7%, which were lower than the other treatments. Again, the normal oral (HGF-1) cells showed very low cytotoxicity to UVC/CPC (91.9% viability). For 72 h viability, the cell viability for the UVC/CPC of HSC-3 and OC-2 cells was 28.6% and 27.0%, respectively, both of which were lower than the other treatments. The normal oral (HGF-1) cells showed low cytotoxicity to UVC/CPC (68.2% viability). Moreover, the synergistic antiproliferation of UVC/CPC in oral cancer cells is higher in 48 and 72 h than 24 h. Therefore, UVC/CPC exhibited preferential and synergistic antiproliferation against oral cancer cells but showed low cytotoxicity to normal oral cells.

### 2.2. Cooperative ROS/Mitochondrial Superoxide (MitoSOX) Generation of Oral Cancer Cells by CPC/UVC Treatment

**Figure 2:**
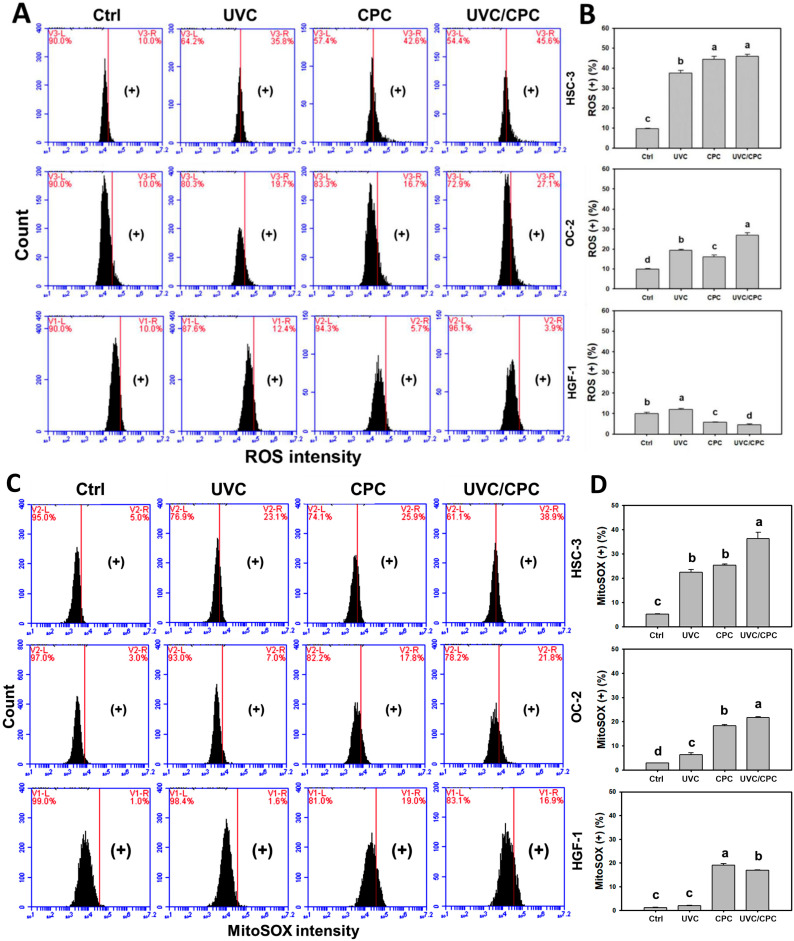
ROS and MitoSOX changes of oral cancer and normal oral cells following UVC and/or CPC treatments. Oral cancer (HSC-3 and OC-2) and oral normal (HGF-1) cells were arranged to treat with vehicle control, UVC (10 J/m^2^), CPC (5 μg/mL), and UVC/CPC (10 J/m^2^ and 5 μg/mL). Flow cytometry was performed after 24 h treatment. ROS (+) and MitoSOX (+) populations were labeled with (+), i.e., high intensity. (**A**) ROS patterns. (**B**) Statistics for (**A**). (**C**) MitoSOX patterns. (**D**) Statistics for (**C**). Data, mean ± SD (*n* = 3). Based on multiple comparisons after Tukey’s HSD Test, different tests labeled with different top letters differ significantly (*p* < 0.05). In the example of (**D**) MitoSOX in HSC-3 cells, UVC and CPC showing “b, b” indicated a non-significant difference between each other. UVC/CPC and CPC in HSC-3 cells showing “a, b” indicated a significant difference between each other.

Following UVC and/or CPC treatments for 24 h, the peaks of ROS and MitoSOX patterns in oral cancer (HSC-3 and OC-2) cells were shifted to the right (high intensity) (Figure 2A,C). The ROS (+) (%) of these two oral cancer cell lines were more elevated in CPC alone as well as in a combined UVC/CPC treatment, than in others (control and UVC) (Figure 2B). In contrast, the ROS levels of single or combined treatments in normal oral cells (HGF-1) showed minor changes (Figure 2A,B).

In addition, the MitoSOX (+) (%) of these oral cancer cells were higher in a UVC/CPC combined treatment than in other treatments (Figure 2D). For normal oral cells (HGF-1), the MitoSOX levels of CPC alone or combined treatments were higher than UVC alone and control (Figure 2C,D). Therefore, either ROS and/or MitoSOX may be cooperatively and differentially induced by UVC/CPC treatment in oral cancer cells.

### 2.3. Cooperative Mitochondrial Membrane Potential (MMP) Depletion and Mitochondrial Mass (Mito Mass) Increase of Oral Cancer Cells by CPC/UVC Treatment

**Figure 3:**
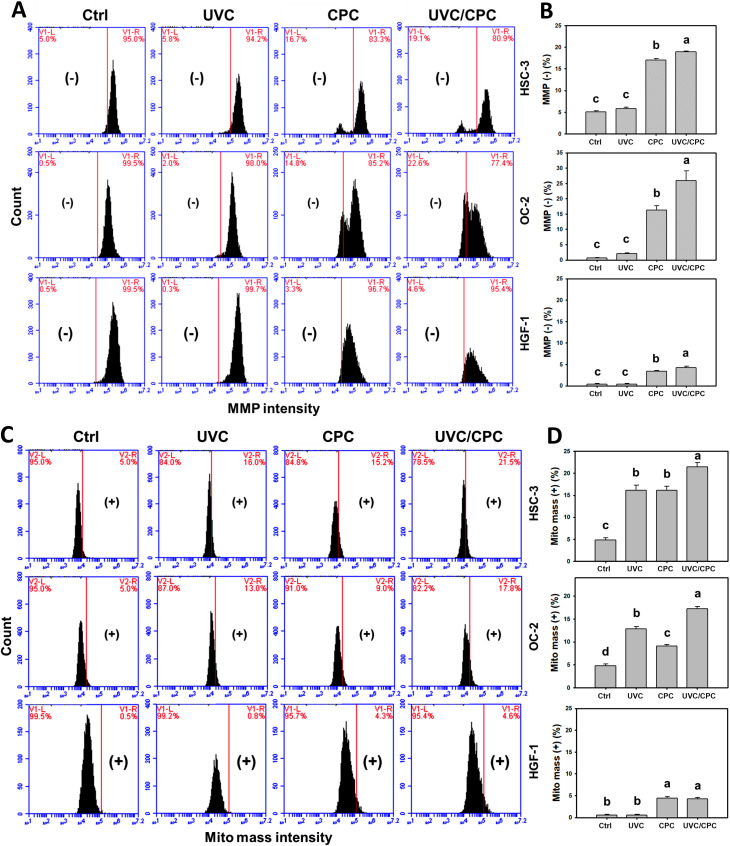
MMP and mitochondrial mass change of oral cancer and normal oral cells following UVC and/or CPC treatments. Oral cancer (HSC-3 and OC-2) and oral normal (HGF-1) cells were arranged to treat with vehicle control, UVC (10 J/m^2^), CPC (5 μg/mL), and UVC/CPC (10 J/m^2^ and 5 μg/mL). Flow cytometry was performed after 24 h treatment. MMP (−) and Mito mass (+) populations were labeled with (+) and (−), respectively, i.e., high and low intensity. (**A**) MMP patterns. (**B**) Statistics for (**A**). (**C**) Mito mass patterns. (**D**) Statistics for (**C**). Data, mean ± SD (*n* = 3). Based on multiple comparisons after Tukey’s HSD Tests, different tests labeled without the same top letters differed significantly (*p* < 0.05). For the example of (**D**) Mito mass in HSC-3 cells, UVC and CPC showing “b, b” indicated a non-significant difference between each other. UVC/CPC and CPC in HSC-3 cells showing “a, b” without overlapping letters indicating a significant difference between each other.

Following UVC or/and CPC treatments for 24 h, the MMP and Mito mass patterns in oral cancer (HSC-3 and OC-2) cells were shown (Figure 3A,C). The MMP (−) (%) of these two oral cancer cell lines was higher in a UVC/CPC combined treatment than in others (control, UVC, or CPC) (Figure 3B). In contrast, the MMP levels of single or combined treatments in normal oral cells (HGF-1) showed minor changes (~5%) (Figure 3A,B).

The Mito mass (+) (%) of these two oral cancer cell lines was higher in a UVC/CPC combined treatment than in other treatments (Figure 3D). In contrast, the Mito mass levels of single or combined treatments in normal oral cells (HGF-1) showed minor changes (~5%) (Figure 3C,D).

### 2.4. Cooperative Cell Cycle Disturbance of Oral Cancer Cells by UVC and CPC/UVC Treatments

**Figure 4:**
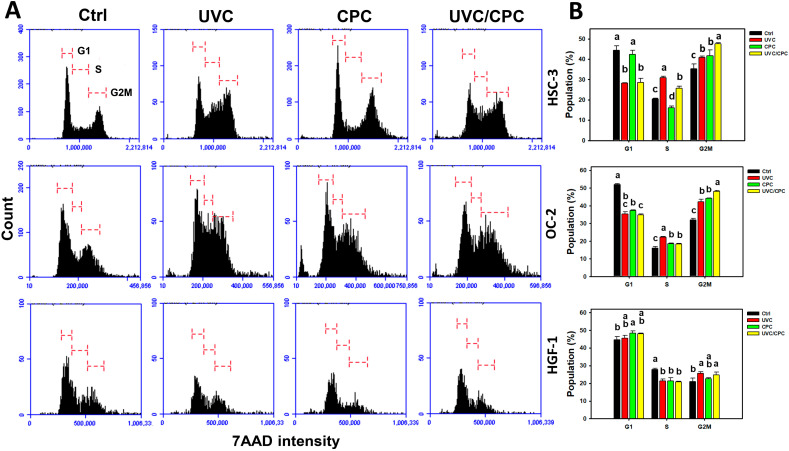
Cell cycle change of oral cancer and normal oral cells following UVC or/and CPC treatments. Oral cancer (HSC-3 and OC-2) cells were treated with vehicle control, UVC (10 J/m^2^), CPC (5 μg/mL), and UVC/CPC (10 J/m^2^ and 5 μg/mL). The cell cycle was determined by flow cytometry after 24 h treatment. (**A**) Cell cycle patterns. (**B**) Statistics for (**A**). Data, mean ± SD (*n* = 3). Based on multiple comparisons after Tukey’s HSD Tests, different top letters indicated significant differences for the same cell cycle phase of the same cell lines (*p* < 0.05).

Following UVC and/or CPC treatments for 24 h, the cell cycle patterns in oral cancer (HSC-3 and OC-2) cells were shown (Figure 4A). Combined treatment (UVC/CPC) in HSC-3 and OC-2 cells showed a higher G2/M accumulation than individual and control treatments (Figure 4B). In contrast, normal oral cells (HGF-1) showed minor changes for each treatment.

### 2.5. Cooperative Apoptosis of Oral Cancer Cells by CPC/UVC Treatment

**Figure 5:**
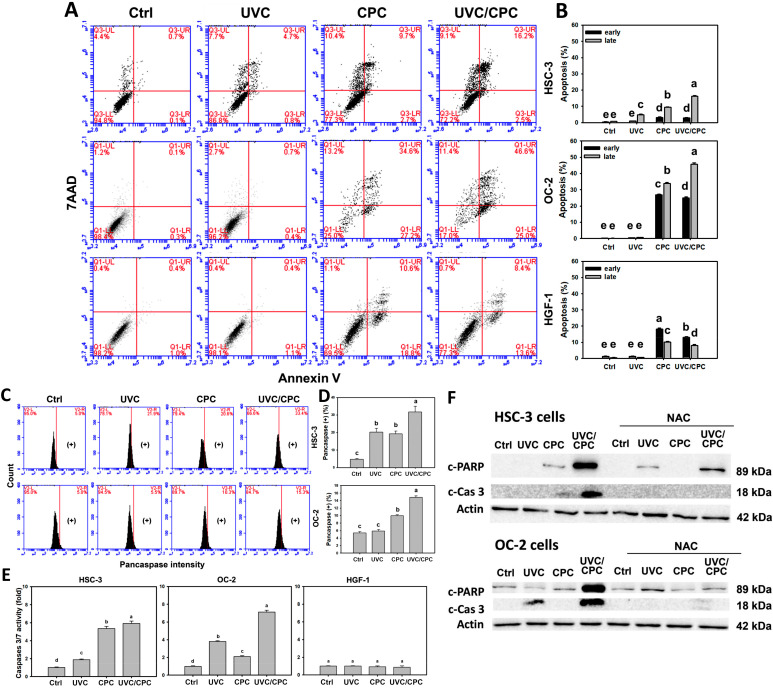
Apoptosis change of oral cancer and normal oral cells following UVC or/and CPC treatments. Cells were arranged to treat with vehicle control, UVC (10 J/m^2^), CPC (5 μg/mL), and UVC/CPC (10 J/m^2^ and 5 μg/mL). Flow cytometry, Cas 3/7 assay, and western blotting were performed after 24 h treatment. (**A**) Annexin V/7AAD patterns. Annexin V (+)/7AAD (−) and Annexin V (+)/7AAD (+) were counted for early and late apoptosis, i.e., + and – for high and low intensity. (**B**) Statistics for (**A**). (**C**) Pancaspase patterns. Pancaspase (+) populations were labeled with (+), i.e., high intensity. (**D**) Statistics for (**C**). (**E**) Cas 3/7 activity. Data, mean ± SD (*n* = 3). Based on multiple comparisons after Tukey’s HSD Tests, different tests labeled without the same top letters differ significantly (*p* < 0.05). (**F**) Western blotting analysis. Apoptotic signaling proteins (c-PARP and c-Cas 3) were detected. Except for NAC pretreatment (4 mM for 1 h), other treatments provided the same results described above.

Following UVC and/or CPC treatments for 24 h, the annexin V/7AAD and pancaspase patterns in oral cancer cells were investigated (Figure 5A,C). The apoptosis-detected annexin V (+) (%) of these two oral cancer cell lines was higher in a combined UVC/CPC treatment than in others (control, UVC, or CPC) (Figure 5B). Moreover, CPC alone or combined treatments in oral cells showed higher late apoptosis than early apoptosis. In contrast, CPC alone or combined treatments in normal oral cells (HGF-1) showed more early apoptosis than late apoptosis (Figure 5A,B).

To further validate that apoptosis took place, the pancaspase (+) (%) of these two oral cancer cell lines was analyzed (Figure 5C). Pancaspase activity was higher in combined UVC/CPC treatment than in others (Figure 5D). UVC/CPC also activates the caspases 3/7 activity in oral cancer (HSC-3 and OC-2) cells but shows no change in normal oral (HGF-1) cells by luminescence detection (Figure 5E).

By western blotting, more complex apoptosis signaling was examined (Figure 5F). It showed that combined UVC/CPC treatment induces cleaved-poly (ADP-ribose) polymerase (c-PARP) and cleaved-caspase 3 (c-Cas 3) expressions contrary to individual and control treatments in HSC-3 and OC-2 cells. To further confirm the contribution of oxidative stress in triggering apoptosis, the oxidative stress inhibitor (*N*-acetylcysteine; NAC) was used. These UVC/CPC combined treatment-induced apoptosis protein expressions were suppressed by a pretreatment with NAC.

### 2.6. Cooperative Expressions of γH2AX and 8-Hydroxy-2’-Deoxyguanosine (8-OHdG) in Oral Cancer Cells by CPC/UVC Treatment

**Figure 6:**
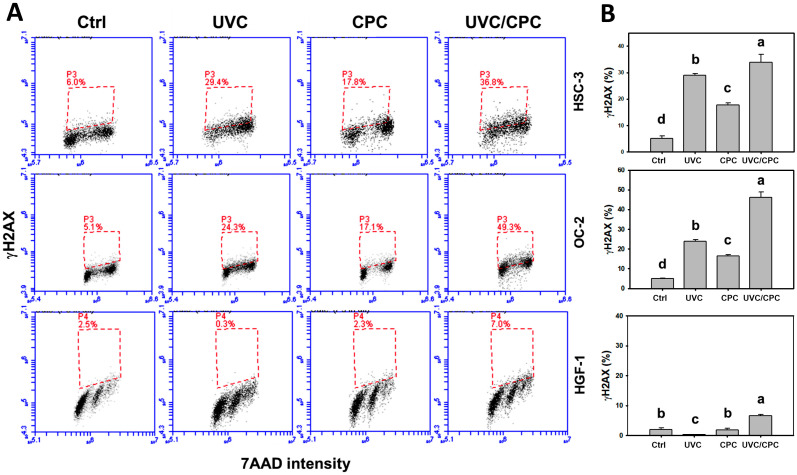

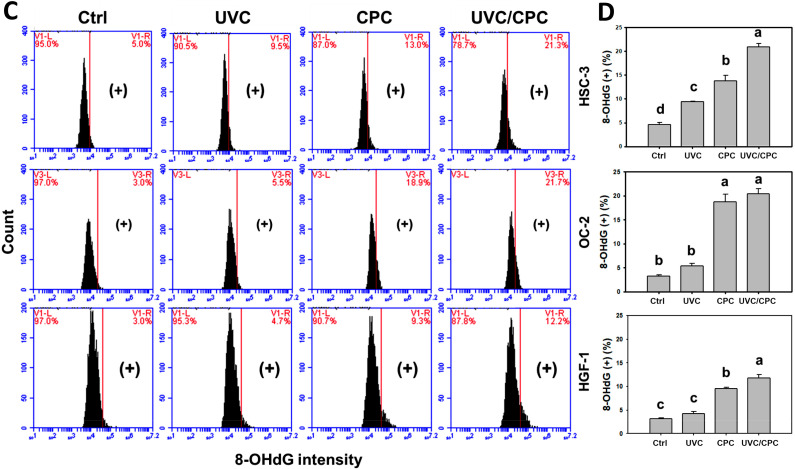
γH2AX and 8-OHdG changes of oral cancer and normal oral cells following UVC and/or CPC treatments. Oral cancer (HSC-3 and OC-2) and oral normal (HGF-1) cells were arranged to treat with vehicle control, UVC (10 J/m^2^), CPC (5 μg/mL), and UVC/CPC (10 J/m^2^ and 5 μg/mL). Flow cytometry was performed after 24 h treatment. γH2AX (+) and 8-OHdG (+) populations were arranged with trapezoid and labeled with (+), respectively, i.e., + for high intensity. (**A**) γH2AX pattern. (**B**) Statistics (**A**). (**C**) 8-OHdG patterns. (**D**) Statistics in (**C**). Data, mean ± SD (*n* = 3). Based on multiple comparisons after Tukey’s HSD Tests, different tests labeling without the same top letters differ significantly (*p* < 0.05).

Following UVC and/or CPC treatments for 24 h, the γH2AX and 8-OHdG patterns in oral cancer cells were shown (Figure 6A,C). The γH2AX (+) (%) of these two oral cancer cell lines was higher in a combined UVC/CPC treatment than in others (control, UVC, or CPC) (Figure 6B). In contrast, the γH2AX levels of single or combined treatments in normal oral cells (HGF-1) showed minor changes (~5%) (Figure 6A,B).

The 8-OHdG (+) (%) of these oral cancers cells was higher in the combined UVC/CPC treatment than in the other treatments (Figure 6D). In contrast, the γH2AX levels of single or combined treatments in normal oral cells (HGF-1) showed minor changes (~12%) (Figure 6C,D). Notably, the 8-OHdG level was higher in oral cancer cells than normal oral cells in single and combined treatments.

## 3. Discussion

The anti-proliferation effect of the combined UVC/CPC treatment of oral cancer cells was validated in the present study. Furthermore, its cooperative anti-proliferation was associated with cell cycle progression, oxidative stress, mitochondrial dysfunction, apoptosis, and DNA damage.

### 3.1. UVC/CPC Provides the Cooperative Antiproliferation of Oral Cancer Cells

At 24 h MTS assay (Figure 1), a CPC/UVC combined treatment (10 J/m^2^ and 5 μg/mL) showed lower viability of oral cancer cells (HSC-3 and OC-2) than normal oral (HGF-1) cells, i.e., 62.0%, 62.1% vs. 79.4%, respectively. For long term observation (Figure 1), this CPC/UVC combined treatment at 48 h (viability 24.2% and 26.6%) and 72 h (viability 28.6% and 27.0%) showed more antiproliferation to oral cancer cells (HSC-3 and OC-2) than 24 h (viability 62.0% and 62.1%). However, normal oral cells (HGF-1) are healthier at 48 h than 24 h and 72 h, i.e., viability 91.9% vs. 79.4% and 68.2%. Concerning drug safety, normal oral HGF-1 cells show low cytotoxic effects following both individual treatments (UVC or CPC) and combined UVC/CPC treatment for 24 h and 48 h. Accordingly, CPC/UVC combined treatment (10 J/m^2^ and 5 μg/mL) at 48 h is the optimal condition for selective antiproliferation in oral cancer cells but low cytotoxicity in normal oral cells.

The optimal conditions and oral cancer cell lines were different between crude methanolic extract of *Cryptocarya concinna* roots (MECCrt) [16] and the present study (MECCrt-derived CPC), which made it hard to compare truly. However, we provided brief information to show the difference between them. MECCrt [16] seems to show better antiproliferation (viability 54.5%) at 24 h treatment for combined treatment (UVC 14 J/m^2^ and MECCrt 10 μg/mL) for oral cancer cells (Ca9-22) than the MECCrt-derived CPC at the condition (UVC 10 J/m^2^ and CPC 5 μg/mL)) for oral cancer cells (HSC-3 and OC-2).

### 3.2. Oxidative Stress Plays an Important Role for Cooperative Antiproliferation of Oral Cancer Cells Treated by UVC/CPC

UVC irradiation can induce ROS generation [9,10,22] and apoptosis [23] in cancer cells and animal models. Some studies reported natural products such as curcumin and genistein could protect against UVC irradiation-induced oxidative stress in cancer cells [24,25]. Alternatively, some anticancer agents that generate exogenous ROS to cancer cells may overload their ROS tolerance and result in cell death [26,27]. Accordingly, a combined UVC and anticancer agents treatment may improve ROS generation compared to individual treatments.

CPC is known to have an ROS generation ability in oral cancer cells [21]. The present study shows that UVC/CPC exhibits higher oxidative stress (ROS and MitoSOX) than a single treatment in oral cancer cells (Figure 2). Moreover, CPC-induced ROS generation of combined treatment was higher in oral cancer (HSC-3 and OC-2) cells than in oral normal (HGF-1) cells. Notably, the ROS levels for CPC alone and UVC/CPC were not significantly different in HSC-3 cells. In contrast, CPC-induced MitoSOX generation of combined treatment was higher in oral cancer (HSC-3) cells than oral cancer (OC-2) and oral normal (HGF-1) cells. These results suggested that CPC-induced ROS generation mainly occurred in cancer cells, while CPC-induced MitoSOX generation is cell type-dependent.

It is possible that the observation time (24 h) is not the optimal condition for ROS and MitoSOX detections in UVC/CPC treatment. This warrants a detailed investigation for the time-course monitoring in the future. Additionally, all UVC/CPC-induced changes may have joint effects to cause cooperative antiproliferation against oral cancer cells.

### 3.3. UVC/CPC Shows the Cooperative Mitochondrial Dysfunction, G2/M Arrest, and Apoptosis in Oral Cancer Cells

Several methods can evaluate mitochondrial function. In addition to MitoSOX, MMP and Mito mass are biomarkers for mitochondrial dysfunction. Oxidative stress induces MMP depletion [28]. Moreover, oxidative stress enhances Mito mass [29], compensating for defective mitochondria [30,31,32]. For example, high-dose hydrogen peroxide enhances Mito mass in osteosarcoma cells [29]. 5 Gy radiation increases Mito mass in human keratinocyte HPV-G cells [31]. Resveratrol increases Mito mass and induces apoptosis in colon cancer SW620 cells [33]. UVC/sulfonyl chromen-4-ones combined treatment in oral cancer cells causes higher Mito mass and triggers apoptosis [10]. Efavirenz triggers apoptosis associated with oxidative stress (MitoSOX generation and MMP depletion) and Mito mass in liver cancer cells [34].

Similarly, the current study demonstrates that UVC/CPC combined treatment induces a higher Mito mass production, apoptosis induction, and MMP depletion than individual treatments (UVC or CPC) in oral cancer cells. Moreover, CPC-induced MMP depletion and Mito mass increment were higher in oral cancer cells than normal oral cells, suggesting that CPC induces selective mitochondrial dysfunction in oral cancer cells rather than normal oral cells. Accordingly, combined phototherapeutic treatment of UVC/CPC generates high oxidative stress, leading synergistically to mitochondrial dysfunction in oral cancer cells.

In addition, cell cycle disturbance may lead to apoptosis. For example, ginsenoside Rf [35], sinularin [36], curcumin [37], and withanolide C [38] induce G2/M arrest, which is frequently associated with apoptosis in cancer cells. Similarly, a combined treatment with UVC/CPC induces higher G2/M arrest than individual treatments (UVC or CPC) in oral cancer cells. In contrast, the cell cycle distribution of normal oral cells (HGF-1) was similar to each treatment. Moreover, apoptosis signaling such as c-PARP and c-Cas 3 was caused by a combined UVC/CPC treatment compared to individual treatments. A NAC pretreatment suppressed such apoptosis signaling. Accordingly, a combined treatment of UVC/CPC induces a higher apoptosis rate in an oxidative stress-dependent manner than individual treatments.

### 3.4. UVC/CPC Causes DNA Damage in Oral Cancer Cells

Oxidative stress is a significant factor in DNA damage induction [39]. UVC caused both γH2AX-detected general DNA damage and 8-OHdG-detected oxidative DNA damage [10]. CPC previously caused γH2AX-detected DNA damage [21]. Additionally, the present study demonstrated that CPC also induced 8-OHdG-detected DNA damage. A combined treatment of UVC/CPC generated high oxidative stress, leading to DNA damage in oral cancer cells. Moreover, CPC-induced γH2AX and 8-OHdG increments were higher in oral cancer cells than normal oral cells, suggesting that CPC induces selective DNA damage in oral cancer cells rather than normal oral cells.

The present study detected γH2AX levels by flow cytometry, but it could not provide direct evidence that γH2AX was targeted at the DNA damage sites, which could be proved by γH2AX foci [40,41]. It warrants detailed experiments for immunofluorescence analysis to evaluate the γH2AX foci changes in UVC and/or CPC-treated oral cancer cells.

### 3.5. Limitations of UVC/CPC Application in Oral Cancer Cells

UVC irradiation may induce photokeratitis for long-term exposure or a high dose rate [42]. However, the present study chose a low dose and cytotoxicity rate of UVC at 70–80% viability to avoid this possibility of side effects. UVC is higher-energy non-ionizing radiation than UVA and UVB. UVC is low penetrating but suitable for surface sterilization and DNA cross-linking [12]. UVC irradiation may potentially kill surface cancer cells such as squamous cell carcinoma (SCC) in situ. Since 90% of oral cancer cells belong to SCC [43], UVC may have the potential for clinical testing. Moreover, the UVC generator device has more flexibility to operate in the oral cavity to cure oral cancer. Recently, several animal studies have reported using UVC as an anticancer application. For example, UVC can inhibit tumor growth for superficial brain tumors in mice [15]. However, the UVC/CPC combined treatment still needs an animal study before clinical testing to prove its in vivo antitumor effects in the future.

Moreover, the UVC dose needs to be adjusted to the different depth layers of the oral tumor tissues. The depth determination for UVC penetration has been reported in excised tumors by detecting the expressions of DNA damage response protein (p53-binding protein 1; 53BP1) [13]. The 53BP1 foci number in the tumors at various depths was analyzed using confocal microscopy. Alternatively, the real penetrability of UVC and the depth of a squamous cell carcinoma may be determined by the diacetylene-based film dosimeters that had been applied for UVB phototherapy [44]. Additionally, this combined treatment may be unsuitable for non-surface-situated tumors due to penetration depth limitations of UVC radiation.

The combined treatment enhanced the cell-killing effects on oral cancer cells; however, it also showed a modest reduction in cancer cell viability. It warrants detailed investigation on survival after longer treatment times (48 or 72 h) to see whether effects are more prominent. Alternatively, the fractional irradiation/CPC combined treatment may be considered to perform for different time intervals in the future.

Although the combined UVC/CPC treatment showed higher viability in normal oral cells (79.4%) than that in oral cancer cells (62%), the changes for oxidative stress, cell cycle disturbance, apoptosis, and DNA damage in normal oral cells warrants detailed investigations compared to these changes in oral cancer cells. Accordingly, the contribution of these changes in UVC/CPC-induced preferential killing of oral cancer cells should be explored.

### 3.6. Possible Targets of UVC/CPC

To predict the possible target of CPC, we used the MetaCore/MetaDrug™ platform [45,46], which uses QSAR models to predict the input of a molecule’s molecular pathway and pharmacokinetic activity. The predicted targets of CPC were the multidrug-resistance gene (MDR1) and cytochrome P450 2D6 (CYP2D6). MDR1 was predicted to be inhibited, and CYP2D6 was expected to be metabolized by CPC. UVC, the non-ionizing radiation, is commonly known to attach macromolecules and cause DNA damage, intracellular protein, and lipid peroxidation [47]. This warrants a detailed investigation of the impact of CPC on the expressions of MDR1 and CYP2D6 in oral cancer cells. Moreover, the potential effects of UVC on CPC itself also need to be examined in the future.

## 4. Materials and Methods

### 4.1. Cell Culture, Reagents, and Cell Viability

Two oral cancer cell lines (HSC-3 from ATCC; Manassas, VA, USA and OC-2 from Dr. Wan-Chi Tsai (Kaohsiung Medical University, Taiwan) [48]) and one normal oral cell line (HGF-1 from the HSRRB; Osaka, Japan) were used. DMEM/F-12 (Dulbecco’s Modified Eagle Medium (DMEM)/F-12) were enriched with standard antibiotics (penicillin and streptomycin) and 10% fetal bovine serum (Gibco; Grand Island, NY, USA). HSC-3 and OC-2 are human tongue and buccal mucosa SCC cell lines, where the HGF-1 is the human gingival cell line.

For oral cancer cells (HSC-3 and OC-2), the cell seeding density for the 6 cm dish at the 24, 48, and 72 h experiments was 5 × 10^4^, 3 × 10^4^, and 10^4^, respectively. For normal oral cells (HGF-1), all the cell seeding densities for the 6 cm dish at 24, 48, and 72 h experiments were 3 × 10^4^, 2 × 10^4^, and 10^4^, respectively. After seeding overnight, cells were treated with UVC and/or CPC for the designed conditions as indicated in the figure legends.

CPC (MW: 282.29 g/mol), the *Cryptocarya concinna* root-derived compound, was prepared according to our previous work [21]. In addition, 24 h cell viability was tested by an MTS assay (Promega Corporation, Madison, WI, USA) and the MTS reaction was read by an ELISA reader at 490 nm [49]. The final concentration of dimethyl sulfoxide (DMSO) for all drug treatments and control was 0.01%. The ROS inhibitor *N*-acetylcysteine (NAC) (Sigma-Aldrich, St. Louis, MO, USA) (4 mM) [50,51,52,53] was pretreated for 1 h to examine the ROS dependence of the improving effect of CPC/UVC combined treatment in oral cancer cells.

### 4.2. ROS and MitoSOX

After harvest and washing, UVC and/or CPC-treated cells were resuspended for ROS and MitoSOX reactions. In brief, ROS was detected after staining at 37 °C for 30 min with 100 nM 2’,7’-dichlorodihydrofluorescein diacetate (H_2_DCF-DA) (Sigma-Aldrich, St. Louis, MO, USA) [54]. MitoSOX was detected after staining at 37 °C for 30 min with 50 nM MitoSOX™ Red (Thermo Fisher Scientific, Carlsbad, CA, USA) [50]. Subsequently, the change of ROS and MitoSOX contents were measured by Accuri C6 flow cytometry (Becton-Dickinson, Mansfield, MA, USA) at the FL1 and FL3 channels (ex: 488 nm; em: 515–545 nm; >650 nm), respectively.

### 4.3. MMP and Mito Mass

After harvest and washing, UVC and/or CPC-treated cells were resuspended for MMP and Mito mass reactions. In brief, MMP was detected after staining at 37 °C for 30 min with 5 nM MitoProbe^TM^ DiOC_2_ (3) (Thermo Fisher Scientific, Carlsbad, CA, USA) [55]. In addition, Mito mass was detected after staining at 37 °C for 30 min with 300 nM of mitochondria localizing dye MitoTracker^TM^ Green FM (Thermo Fisher Scientific, Carlsbad, CA, USA) [56]. Subsequently, the MMP and Mito mass changes were measured by flow cytometry at the FL1 channel (ex: 488 nm; em: 515–545 nm).

### 4.4. Cell Cycle Phase

After harvest and washing, UVC and/or CPC-treated cells were resuspended for fixation. Then, cellular DNA was detected after staining at 37 °C for 30 min with 1 μg/mL 7-aminoactinomycin D (7AAD) (Biotium Inc., Hayward, CA, USA). Subsequently, the change of cellular DNA content was measured for determining different cell cycle phases by flow cytometry at the FL3 channel (ex: 488 nm; em: >650 nm) [57].

### 4.5. Annexin V/7AAD-, Pancaspase-, and Caspases 3/7-Detected Apoptosis

After harvest and washing, UVC and/or CPC-treated cells were resuspended for Annexin V/7AAD and pancaspase reactions. Following the manufacturer’s instructions, apoptosis was analyzed by flow cytometry using an annexin V/7AAD mixed kit (Strong Biotech Corp, Taipei, Taiwan). Both annexin V and 7AAD signals were detected by both FL1 and FL3 channels of the Accuri C6 flow cytometer [58].

In addition, in caspases 1, 3, 4, 5, 6, 7, 8, and 9, activation-detected apoptosis were found after incubation at 37 °C for 30 min with a pancaspase assay kit (Abcam, Cambridge, UK) [49]. Subsequently, the change of pancaspase activity was measured by flow cytometry at the FL1 channel (ex: 488 nm; em: 515–545 nm).

For caspases 3/7 activity assay [10], cells were seeded in 6 cm dishes. After UVC and/or CPC treatment, the cell medium (100 μL) was transferred to the 96-well plate. Caspase-Glo^®^ 3/7 kit (Promega; Madison, WI, USA) was applied following the manufacturer’s instructions. After adding the Cas 3/7 reagent (20 μL) to the medium in the 96 well, this reaction needed to stay in darkness at 37 °C for 30 min. Finally, the signals were detected by a microplate luminometer (Berthold Technologies GmbH & Co., Bad Wildbad, Germany).

### 4.6. Western Blot-Detected Apoptosis

25 μg extracted proteins were loaded for SDS-PAGE electrophoresis. They were transferred to the PVDF membrane for antibody reactions as follows. Primary antibodies against the cleaved forms of apoptosis signaling proteins were applied, e.g., c-PARP and c-Cas 3 (Cell Signalling Technology Inc.; Danvers, MA, USA). In addition, the β-actin antibody (Sigma-Aldrich; St. Louis, MO, USA) was selected as an internal control. Other detailed information for western blotting was mentioned, as described previously [59].

### 4.7. γ H2AX- and 8-OHdG-Detected DNA Damages

The biomarker for general DNA damage, γH2AX, was analyzed using antibody detection by flow cytometry [60]. After harvest and washing, UVC and/or CPC-treated cells were resuspended for fixation. Then, cells were incubated at 4 °C for 1 h with a primary antibody against p-Histone H2A.X (Ser 139) (Santa Cruz Biotechnology; Santa Cruz, CA, USA) (1:500). Later, the Alexa 488-modified secondary antibody was applied, and 5 μg/mL 7AAD was used for double staining (30 min, 37 °C). Finally, the γH2AX and 7AAD intensities were measured by flow cytometry at the FL1 and FL3 channels (ex: 488 nm; em: 515–545 nm; >650 nm).

DNA damage with the oxidative adduct 8-OHdG was analyzed using its antibody detection by flow cytometry [61]. After harvest and washing, UVC and/or CPC-treated cells were resuspended for fixation. Then, cells were incubated at 4 °C for 1 h with one-step primary antibody-modified with FITC against 8-OHdG (Santa Cruz Biotechnology, Dallas, TX, USA) (1:10,000) and measured by flow cytometry (Accuri™ C6) at the FL1 channel (ex: 488 nm; em: 515–545 nm).

### 4.8. Statistics

All data were presented as means ± SD (derived from triplicates). One-way ANOVA determined the significant difference among multiple comparisons after Tukey’s HSD Tests [21]. Data showing no overlapping letters differ significantly. Several examples were provided in the figure legends to demonstrate the results of multiple comparisons.

## 5. Conclusions

Combining a radiation source with radiosensitizing chemicals is a common strategy to improve oral cancer radiotherapy [6,7]. But radiosensitizers may equally act on normal tissue and generate potential cytotoxic side effects. Radiosensitizers showing the preferential killing of cancer cells compared to normal cells may solve this problem. In the present study, a preferential killing of the natural product CPC [21] was evaluated for its usefulness against oral cancer cells in combined phototherapy with UVC irradiation compared to individual radiotherapy with UVC only. We demonstrated that a combined treatment of UVC/CPC inhibits oral cancer cell proliferation substantially compared to individual or control treatments. In addition, UVC/CPC exerts preferential anti-proliferation to oral cancer cells compared to normal oral cells.

The improving anti-proliferation mechanism of UVC and CPC was pronounced in a combined treatment rather than at individual treatments. UVC/CPC treatment in combination causes higher oxidative stress, G2/M cell cycle arrest, DNA damage, and apoptosis in oral cancer cells. Therefore, CPC is a potential UVC sensitizing natural product that enhances the preferential killing effect on oral cancer cells.

## Data Availability

Data are contained within the article.
